# Trehalose improves traumatic brain injury-induced cognitive impairment

**DOI:** 10.1371/journal.pone.0183683

**Published:** 2017-08-24

**Authors:** Stuart D. Portbury, Dominic J. Hare, David I. Finkelstein, Paul A. Adlard

**Affiliations:** 1 The Florey Institute of Neuroscience and Mental Health, The University of Melbourne, Parkville, Victoria, Australia; 2 University of Technology Sydney, Elemental Bio-imaging, Sydney, Australia; Louisiana State University Health Sciences Center, UNITED STATES

## Abstract

Traumatic brain Injury (TBI) is a significant cause of death and long-term disability for which there are currently no effective pharmacological treatment options. In this study then, we utilized a mouse model of TBI to assess the therapeutic potential of the stable disaccharide trehalose, which is known to protect against oxidative stress, increase levels of chaperone molecules and enhance autophagy. Furthermore, trehalose has demonstrated neuroprotective properties in numerous animal models and has been proposed as a potential treatment for neurodegeneration. As TBI (and associated neurodegenerative disorders) is complicated by a sudden and dramatic change in brain metal concentrations, including iron (Fe) and zinc (Zn), the collective accumulation and translocation of which has been hypothesized to contribute to the pathogenesis of TBI, then we also sought to determine whether trehalose modulated the metal dyshomeostasis associated with TBI. In this study three-month-old C57Bl/6 wildtype mice received a controlled cortical impact TBI, and were subsequently treated for one month with trehalose. During this time animals were assessed on multiple behavioral tasks prior to tissue collection. Results showed an overall significant improvement in the Morris water maze, Y-maze and open field behavioral tests in trehalose-treated mice when compared to controls. These functional benefits occurred in the absence of any change in lesion volume or any significant modulation of biometals, as assessed by laser ablation inductively coupled plasma mass spectrometry. Western blot analysis, however, revealed an upregulation of synaptophysin, doublecortin and brain derived neurotrophic factor protein in trehalose treated mice in the contralateral cortex. These results indicate that trehalose may be efficacious in improving functional outcomes following TBI by a previously undescribed mechanism of action that has relevance to multiple disorders of the central nervous system.

## Introduction

Traumatic Brain Injury (TBI) is a major global health problem [1] and represents the leading cause of mortality and disability in high-income countries [2]. It is estimated that 1.7 million TBI’s occur annually in the United States alone, and in 2006, 5.3 million people were living with long term disabilities as a consequence of brain injury [3]. The causes of TBI are etiologically diverse, but largely comprise of motor vehicle accidents, occupational hazards, physical violence and falls [3], all of which contribute to a significant public health burden. Additionally, brain trauma incurred through contact combat sports [4,5] and American football [6–8] has been shown to culminate in subsequent behavioral disabilities [9] and pathological effects in the form of chronic traumatic encephalopathy (CTE) [10–12]. Similarly, military blast-induced TBI has also been shown to result in long-term cognitive deficits in affected individuals [13]; over 200,000 U.S. service members deployed to Central Asia and the Middle East have been officially diagnosed with TBI since 2003 [14]. Regardless of the globally increasing incidence of TBI, and despite much effort, there currently is no therapeutic option available to ease or prevent some of the most debilitating symptoms that occur following TBI, such as cognitive dysfunction.

The injured brain is characterized by a number of features, including perturbed metal homeostasis [15–19] and associated oxidative stress [20,21]. Specifically, the biological transition metals iron (Fe) [17,18,22], copper (Cu) [18,23], and zinc (Zn) [16,24–28] have been implicated as having critical roles in TBI. Moreover, these metals have also been proposed to have integral roles in the pathogenesis of neurodegenerative disorders such as Alzheimer’s disease (AD) [29–31], Parkinson’s disease (PD) [32] and Amyotrophic Lateral Sclerosis (ALS) [33], conditions which may all be predated by a history of TBI [34–36]. Given the disruptions that occur in metal homeostasis following TBI, and the associated implications for neurodegeneration, we hypothesized that a compound that was effective at intervening in the neurodegenerative cascade would be of benefit in a TBI model and may also directly or indirectly impact metal ion homeostasis to further improve functional outcomes post-injury. One such candidate is trehalose, a naturally occurring alpha-linked dissacharide composed of two molecules of glucose, that has been shown to protect against oxidative stress [37,38], upregulate growth factor expression and secretion [39], prevent protein aggregation [40–43], and subsequently delay the progression of neurodegeneration in several transgenic mouse models [44–48]. We therefore sought to determine the therapeutic efficacy of trehalose in a mouse TBI model, and to also investigate whether it had any impact on the biometal aberrations observed in TBI.

## Methods

All procedures were carried out in accordance with protocols approved by the Howard Florey Animal Ethics Committee and were conducted in accordance with the Australian Code of Practice for the Care and Use of Animals for Scientific Purposes as described by the National Health and Medical Research Council of Australia.

### Trehalose

Trehalose (Sigma) has a generous safety profile in rodents and humans [49,50] and was posted as “Generally Regarded as Safe” (GRAS) for human consumption by the U.S. Federal Drug Administration (FDA) in October 2000. It is used as a food additive, and is also an excipient in many pharmaceuticals, making trehalose a safe, natural and pharmaceutically accepted product. In this study, it was supplied as a 2% solution in SSV (SSV; 0.9% NaCl, 0.5% Na-carboxymethylcellulose, 0.5% benzyl alcohol and 0.4% Tween 80) via oral gavage.

### Animals and surgical procedures

The surgical procedures and controlled cortical impact (CCI) injury model have been previously described [17]. Briefly, 3-month-old male C57Bl/6 mice were anesthetized via intraperitoneal injection of 100mg/kg ketamine and 10mg/kg xylazine. A 10mm mid-line incision was made over the skull, and the skin and fascia were reflected to make a 4mm craniotomy on the central aspect of the right parietal bone using a motorized drill. Animals were then positioned in a stereotaxic frame and a CCI injury subsequently delivered (3m/s velocity with a 1.5mm penetration depth).

Three groups of animals were subjected to TBI in separate experiments. The first group of animals were treated daily with either trehalose or standard suspension vehicle (SSV) via oral gavage for a period of 31 days after both groups received a TBI. The dosing regimen commenced 24 hours post-TBI. The second group of animals underwent an identical dosing procedure but were treated with either SSV or maltose as a disaccharide control for trehalose, after TBI. A sham animal group, which underwent identical procedures up to and excluding the administration of a TBI, was also included in the second TBI group.

A third group of animals consisting of a trehalose treated and an SSV treated group that did not undergo any behavioral assessment but underwent the same dosing regime, was used for LA-ICPMS metal analysis. There was no maltose or sham group for the LA-ICPMS analysis.

Mice were obtained from the Animal Resource Centre (Murdoch, WA, Australia), and were humanely culled via IP injection of Lethabarb (Virbac, Australia).

### Behavioral assessment

The Morris Water Maze (MWM) was used to assess the effect of trehalose on spatial learning and memory function following TBI. The pre-training acclimation day of the water maze was performed on day 23 of dosing, followed by six days of place discrimination training of four 90 second trials per day, conducted on days 24–29 of dosing. The probe trial was performed 24 hours after training on dosing day 30 to assess retention of the task. Data was processed using the Ethovision automated tracking system prior to statistical analysis.

The open field assessment was performed on day 19 of dosing. The mice were removed from their home cage and placed individually into clear Perspex tracking arenas (Coulburn TruScan, U.S.A.). The total amount of movements over a 60 minute period were recorded and analyzed. The locomotor cells measured the total time in movement in the floor plane by the interruption of a grid of beams.

Y-maze assessment was performed on day 21 of dosing. Three identical arms of the maze were randomly designated start arm, novel arm, and other arm. The Y-maze tests consisted of 2 trials separated by a 1 hour inter-trial interval. The first trial (training) was for 10 minutes, and the mice were allowed to explore only 2 arms (starting arm and other arm). For the second trial (retention), mice were placed back in the maze in the same starting arm, and allowed to explore for 5 minutes with free access to all three arms. By using a ceiling mounted CCD camera, all trials were analyzed for the number of entries the mice made into each arm. Data were expressed as the percentage of novel arm entries made during the retention trial.

### Laser ablation inductively coupled plasma mass spectrometry

A comprehensive description of the laser ablation-inductively coupled plasma mass spectrometry (LA-ICPMS) procedure has been previously published [17]. Briefly, Trehalose treated and vehicle-treated animals (n = 5 per group) were euthanized at 24 hr, 72 hr, 7 day, 14 day and 28 day-post TBI surgeries. Brain tissue was prepared as previously described and was subjected to LA-ICPMS.

### Western blot analysis

The cortex and hippocampus of both ipsilateral and contralateral hemispheres from animals subject to behavioral analysis were homogenized in 15 volumes of ice-cold PBS containing Complete Protease Inhibitor Cocktail tablets (Roche Applied Science, Indianapolis, IN, USA) and subsequently centrifuged (100,000xg) for 30 minutes at 4°C. The supernatant was removed to yield the soluble fraction (S1), whilst the remaining pellet underwent further extraction via 30 minutes of vigorous agitation in the above-mentioned homogenization buffer containing 2% (vol/vol) Triton X100. Insoluble material was pelleted via centrifugation (20,000xg) for 20 minutes, and the supernatant was retained as the membrane fraction (P1). Protein concentrations were determined using Pierce BCA protein assay (Pierce Biotechnology, Rockford, IL, USA) to ensure equal protein loading (10μg) on the gels. Samples were prepared for PAGE by the addition of 4x protein sample loading buffer (LICOR, Lincoln, Nebraska, USA) and 10x NuPAGE sample reducing agent (to a final 1x concentration). Samples were heated to 70°C for 10 min, loaded onto Bolt 4–12% Bis-Tris Plus gels (Invitrogen-Life Technologies, Grand Island, NY, USA) along with Odyssey One-Color protein molecular weight markers (LICOR, Lincoln, Nebraska, USA, Cat LCR928-4000) and run at 125 V for 60 min in appropriately diluted Bolt MES SDS 20x running buffer (Invitrogen-Life Technologies, Grand Island, NY, USA). Gels were transferred to Immobilon–P, PVDF membrane (Millipore) using the Invitrogen Bolt wet-gel Transfer Device (Invitrogen-Life Technologies, Grand Island, NY, USA) at 15 V for 60 min in appropriately diluted 20x Bolt transfer buffer (Invitrogen-Life Technologies, Grand Island, NY, USA). Membranes were blocked in tris-buffered saline with tween 20 (TBST) containing 5% skim milk powder and then incubated with primary antibody overnight at 4°C (Doublecortin (DCX) antibody diluted 1:1000, Cell Signaling Technology, Danvers, MA, USA Cat # 4604; Synaptophysin antibody diluted 1:1000, Millipore, MA, USA, Cat # AB9272; BDNF antibody diluted 1:5000, ABCAM Cambridge MA, USA, Cat # 108319; Pro-BDNF antibody diluted 1:1000, Saphire Biosciences, Redfern NSW, Australia, Cat # R-087-100; GAPDH diluted 1:10000 Millipore, MA, USA, Cat # MAB374). Blots were rinsed in TBST and incubated with appropriate secondary antibody at room temperature for 1 hour (IRDye800 Goat anti-mouse Cat # LCR926-32210, IRdye800 Goat anti-Rabbit Cat # LCR926-32211, IRDye680 Goat anti-mouse Cat # LCR926-68070, IRDye680 Goat anti-Rabbit Cat # LCR926-68071. LI-COR Biosciences, Lincoln, Nebraska, USA), followed by further rinsing and imaged using a LI-COR Odyssey Imaging system (LI-COR Biosciences, Lincoln NE, USA), and analysed with Image Studio Lite software (LI-COR Biosciences, Lincoln NE, USA). Sample data were normalized to total protein loaded and to the GAPDH or β-actin loading control.

### Statistical analysis

**Fig 1:**
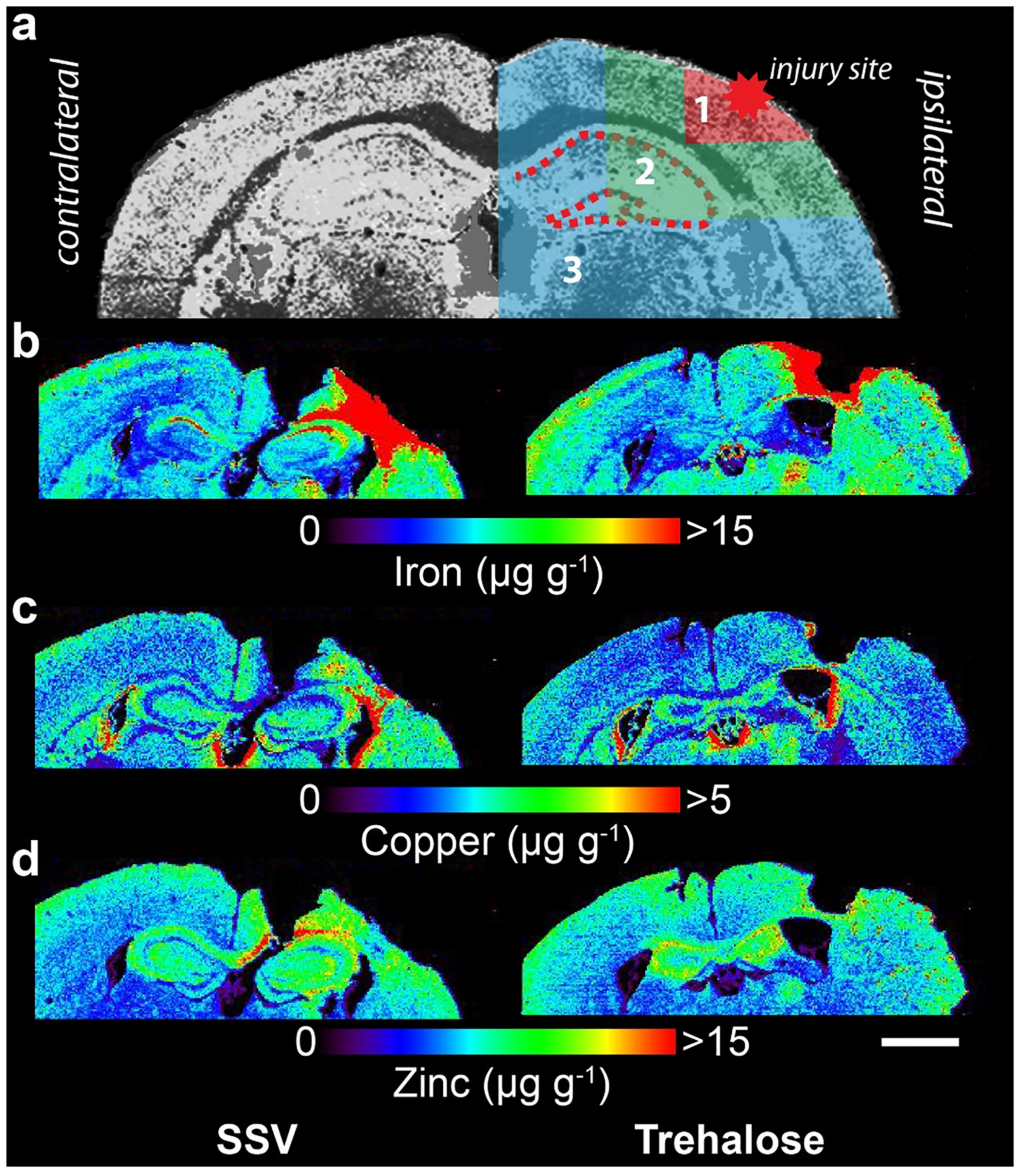
Representative LA-ICPMS schematic for iron, copper and zinc. a) Schematic demonstrating regions of interest selection for assessment of changes in metal levels radial to the site of impact overlaid the copper distribution of an unlesioned, untreated brain. b-d) Representative 28 day LA-ICP-MS images of the upper two quadrants of both hemsipheres for iron, copper and zinc. Selected ROIs were applied to each measured metal and extracted for statistical comparisons. Whole-hemisphere metal concentrations and equivalent ROIs on the contralateral side were also extracted. Images of sections were taken at approximately bregma +2.7 mm from 24 hours to 28 days post-lesion. All images have a lateral spatial resolution of 30 μm. Scale bar = 1 mm.

For LA-ICPMS samples, statistical analysis was carried out in Prism 7 (Graph-Pad) software. Where appropriate, analysis was carried out with a two-tailed *t* test with the level of significance set at *p* = 0.05. For multiple comparisons, repeated measures two-way ANOVA with post-hoc Bonferroni’s multiple comparison test was used to assess the Morris water maze, and a two-way ANOVA with post hoc Bonferroni’s analysis was used to identify significant differences between ipsilateral and contralateral regions of interest (ROI’s) “Fig 1”. For western blot analysis, images were produced using Image Studio Lite (version 4.0.21, LI-COR Biosciences) and subsequent statistical analysis was carried out in Prism 7 (Graph-Pad). All statistical analysis on behavioral data was performed using Prism 7 (Graph-Pad).

## Results

### Behavioural analyses

#### Trehalose restores water maze performance following TBI

**Fig 2:**
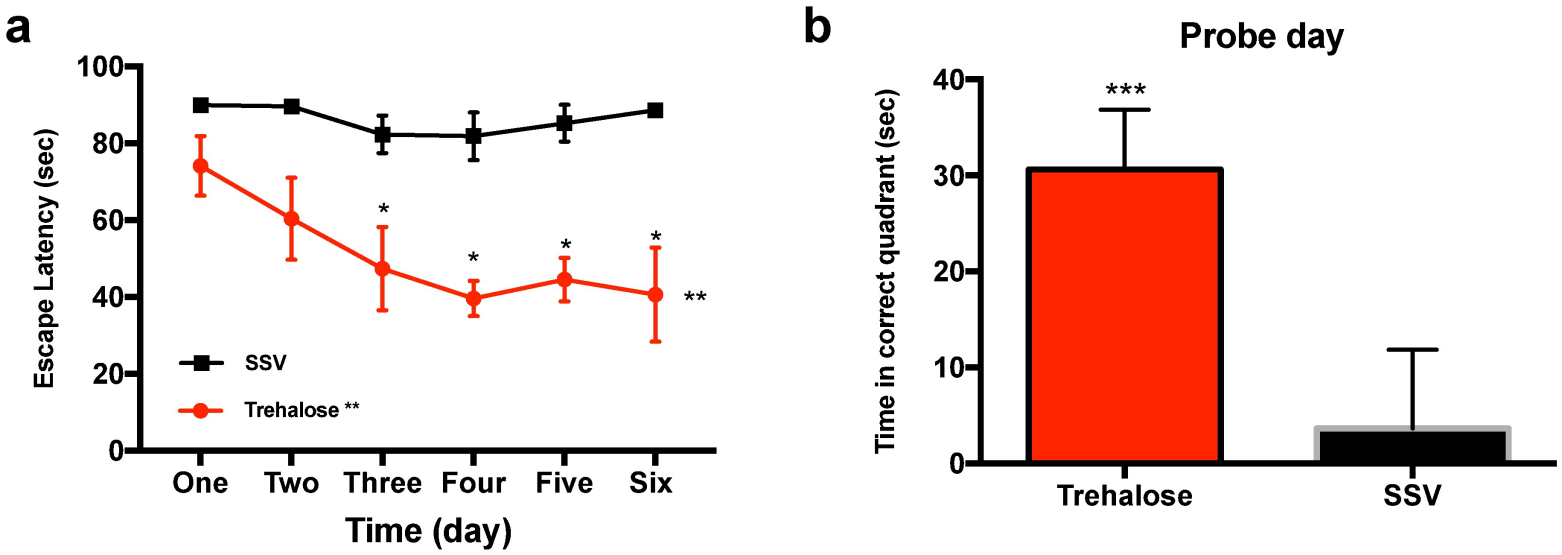
Trehalose improves performance in the Morris water maze. Mice treated post-injury with trehalose revealed an overall significant performance improvement (two-way repeated measures ANOVA **p = 0.0048) in the learning (a) component of the Morris water maze. A *post hoc* Bonferroni’s multiple comparison test confirmed analysis showing significant performance of trehalose treated animals on day three (*p = 0.0200), day four (*p = 0.0197), day five (*p = 0.0038) and day 6 (*p = 0.0092). Trehalose treated mice also performed significantly better than SSV treated litter mates on the recall (b) component of the Morris water maze (*** *p* = 0.0010). Subsequent water maze with maltose treated and sham animals revealed no such significant improvement in the learning component of the trial. However, the recall component revealed a significantly better performance for the uninjured sham animals (ANOVA **p = 0.0019).

Trehalose treated mice (n = 5) demonstrated both an overall improved acquisition of the task (Two-way repeated measures ANOVA *p* < 0.0048) and an improved recall of the task (probe trial, p = 0.0010) as compared with SSV–treated controls (n = 8) “Fig 2a and 2b”. The TBI SSV treated group did not appear to learn during the six-day learning phase of the watermaze, which may be reflective of the deficit in cognition without treatment. In the second TBI group there was no effect of the maltose control (n = 7) on either the learning or the probe trial as compared to SSV-treated controls (n = 13). Similarly, sham mice did not show significant improvement during the learning phase of the trial, however they performed significantly better on the probe trial (ANOVA **p = 0.0019), when compared to maltose and SSV control groups (data not shown).

#### Trehalose increases spontaneous activity in the open field assessment

**Fig 3:**
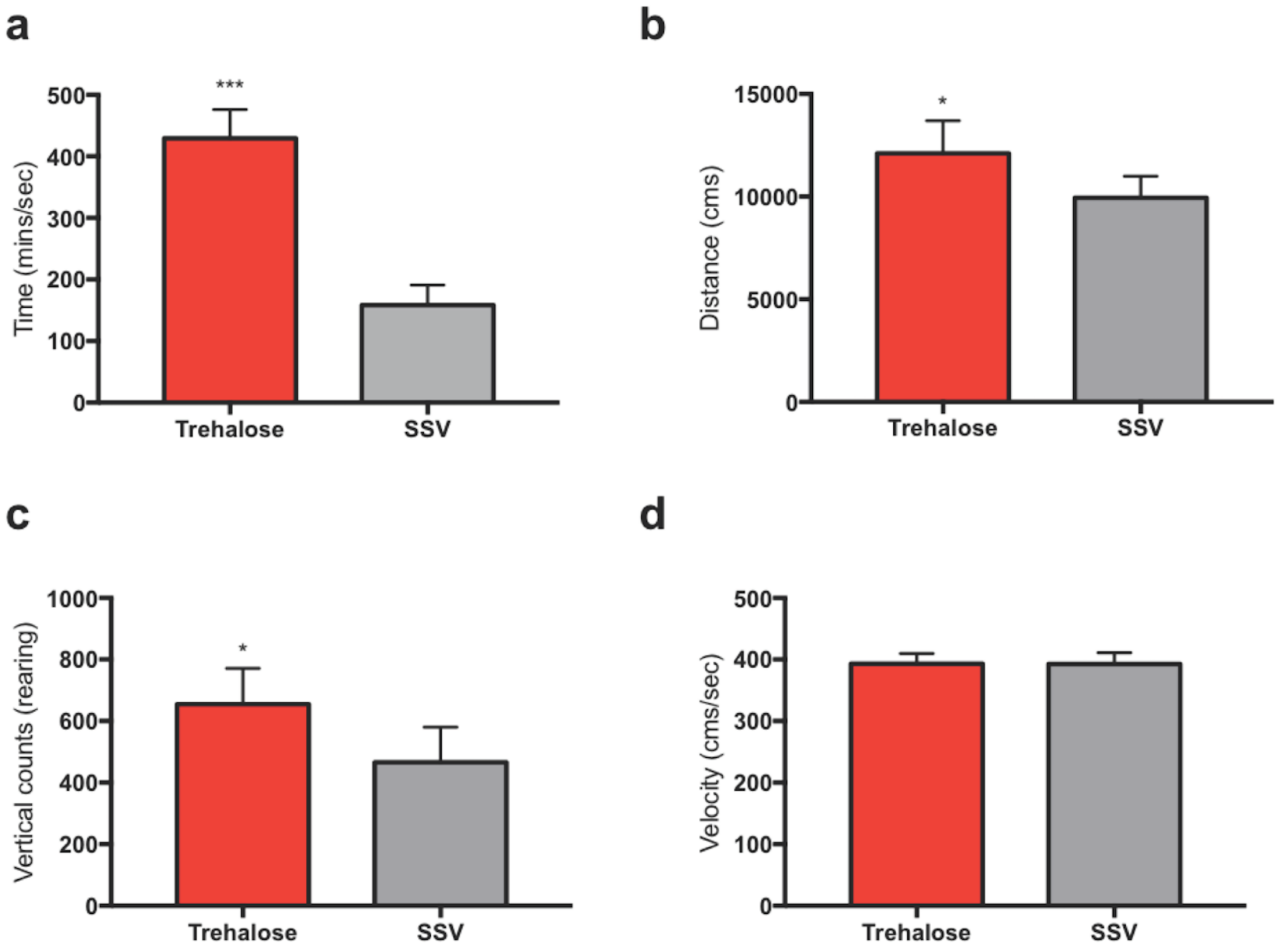
Trehalose treated mice show significantly enhanced willingness to explore in the open field activity test. Post-TBI treated mice showed a significant reduction in anxiety and an increased willingness to explore when compared to SSV littermates. Post-treated mice showed a significant increase in ambulatory time (****p< 0.00001) (a), ambulatory distance (*p< 0.01) (b), vertical counts (*p< 0.01) (c) without a significant increase in velocity (d).

Open field behavioral assessment indicated a significant increase in exploration activity for the trehalose-treated group compared to controls, as exemplified by ambulatory time (*P* < 0.001) “Fig 3a”, total ambulatory counts (*P* < 0.01) “Fig 3b” and vertical counts (*P* <0.01) “Fig 3c”. Importantly, there was no significant difference in ambulatory velocity between the two groups “Fig 3d”. The secondary control group consisting of maltose, SSV and sham mice were not tested in the open field assessment.

#### Trehalose enhances Y-maze performance following TBI

**Fig 4:**
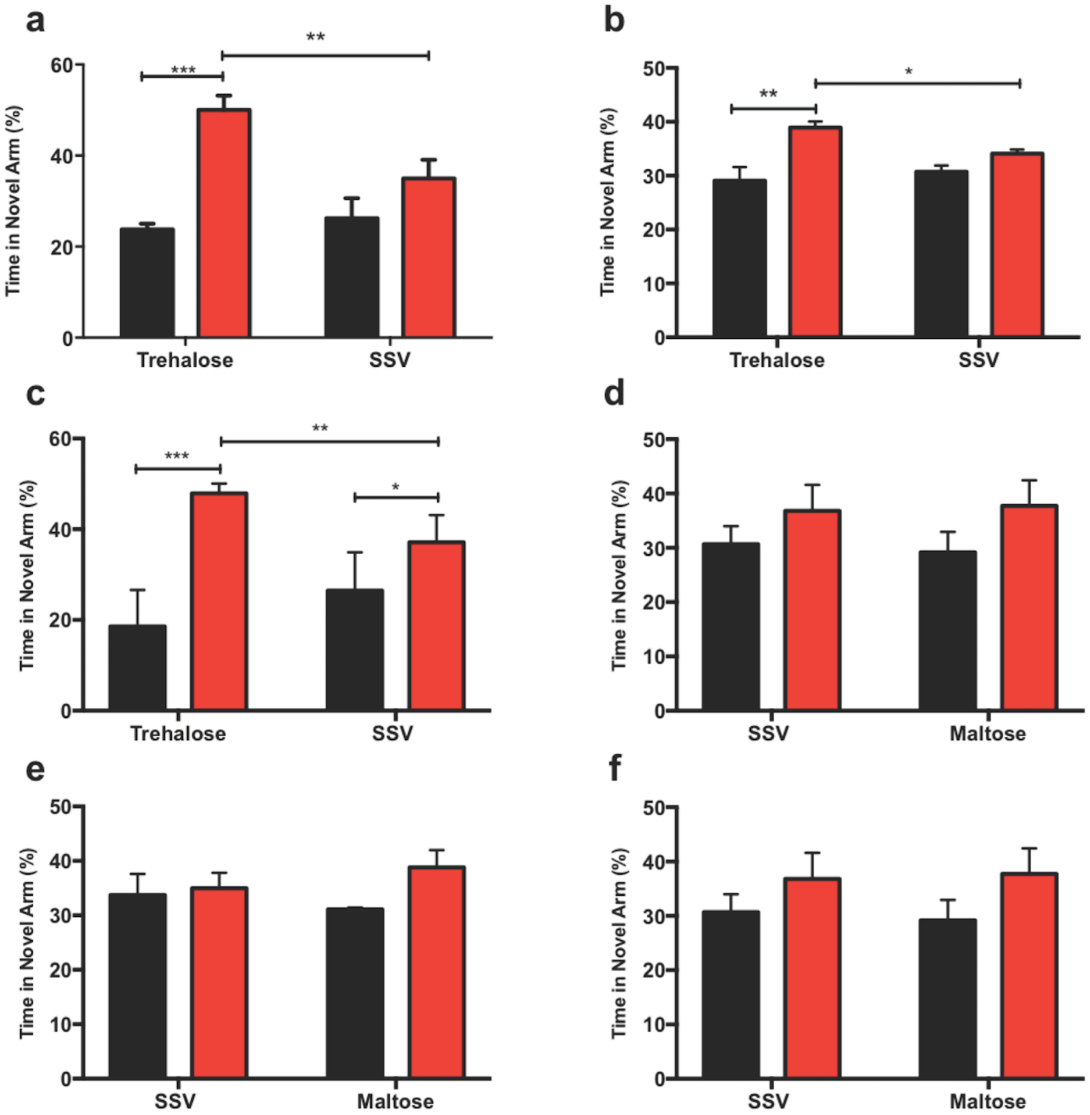
Trehalose treatment improves y-maze performance in TBI mice. Trehalose treated mice showed a significant overall frequency of visitation preference for the novel arm (red) over the start arm (black) compared to SSV treated littermates at both one-minute (a) (**p< 0.001), and five-minute frequency (b) time points (*p< 0.01). Additionally, trehalose treated mice spent a significantly greater duration of time in the novel arm at the one-minute (c) (**p< 0.001) time point when compared to SSV controls. Subsequent y-maze revealed that maltose treated mice had no significant preference for the novel arm at both the one-minute (d) or five-minute (e) frequency measurements when compared to SSV treated littermates. Similarly, the one-minute duration measurement for maltose also revealed no significant increase in the duration time of visits when compared to SSV control littermates.

In the y-maze, the trehalose-treated mice showed an overall significant improvement in both the duration and frequency of visits into the novel arm, indicating a willingness to explore a new environment. One-minute frequency was significantly enhanced over the SSV group (*p* < 0.001) “Fig 4a” as was the five-minute frequency (*p* < 0.01) “Fig 4b”. Additionally, trehalose treated mice spent a significantly increased amount of time in the novel arm at the one-minute time point (*p* < 0.001) “Fig 4c”. There was no effect of the maltose control group on any of the parameters “Fig 4d–4f”.

### Metal analyses

#### Trehalose does not alter zinc levels after TBI

**Fig 5:**
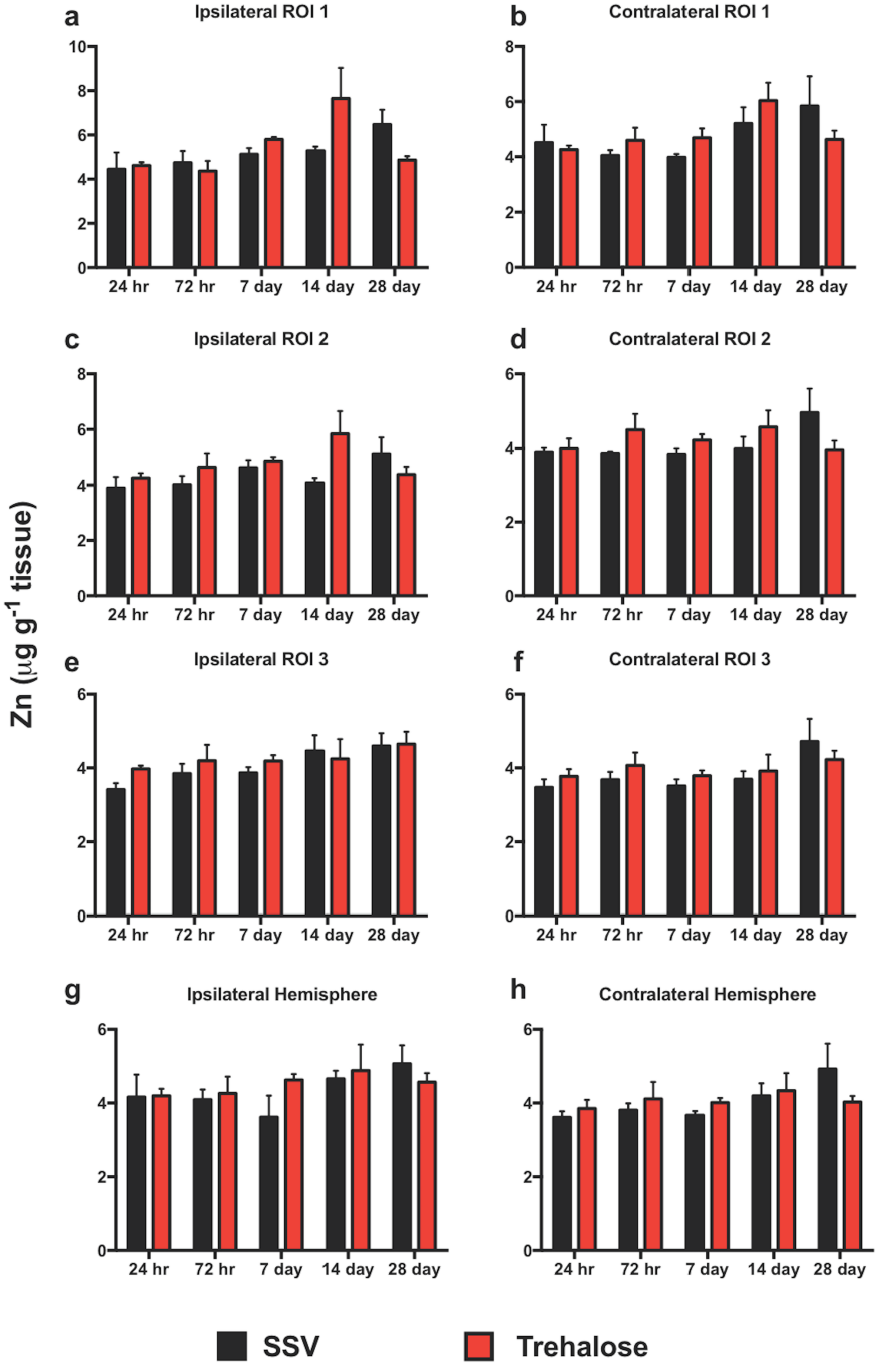
Zinc is unaltered across the timecourse. There were no observed significant increases in Zn in any region analyzed or intraday comparison between trehalose treated and SSV controls.

LA-ICPMS revealed no significant overall change in Zn concentration between trehalose and SSV treatment groups in any region examined in either the ipsilateral or contralateral cortex as assessed by two-way ANOVA and Bonferroni *post hoc* analysis over the time course. Neither were there any significant intra-day changes in Zinc between the trehalose and SSV treatment groups “Fig 5”.

#### Trehalose alters copper concentration after TBI

**Fig 6:**
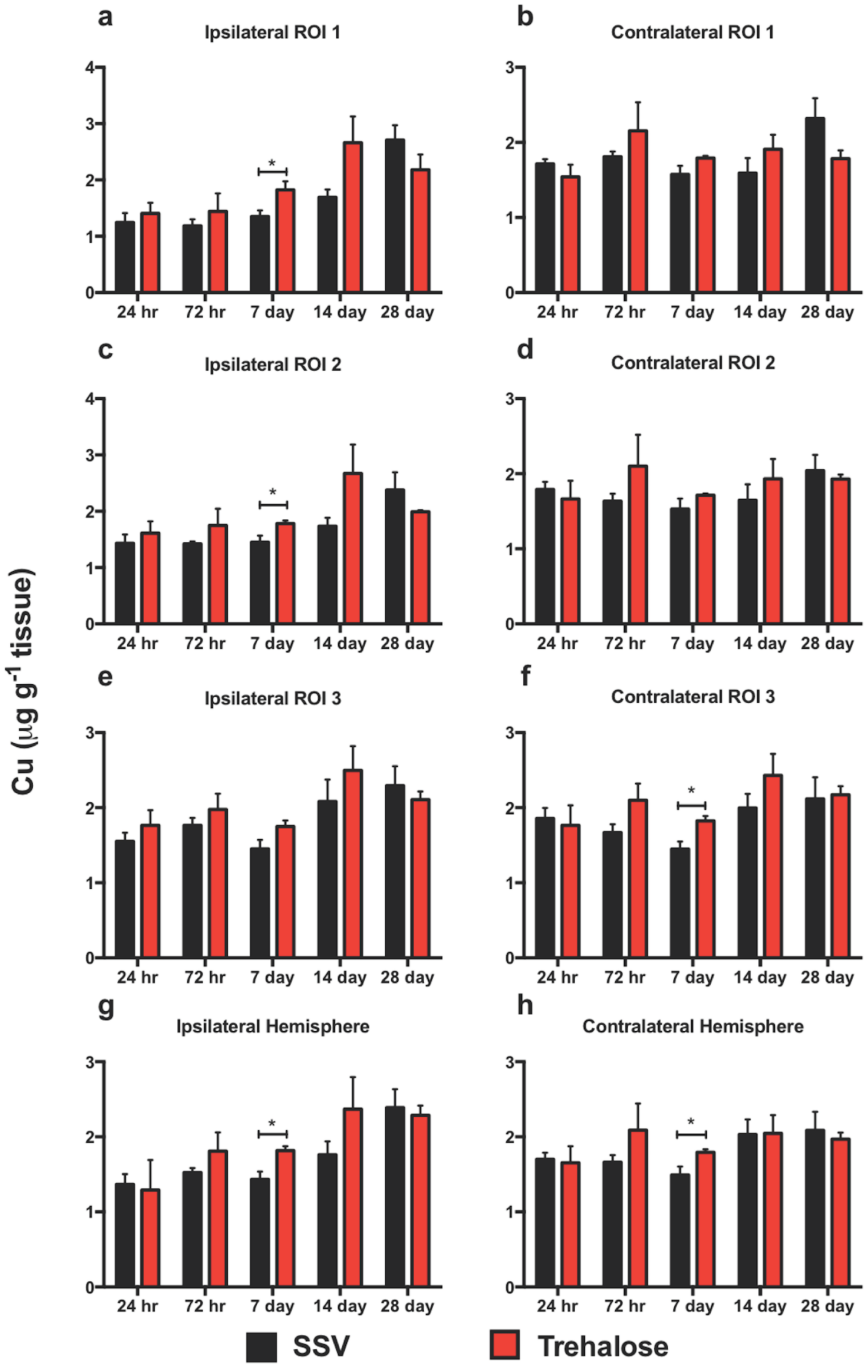
Copper is altered across the time course. Cu revealed ipsilateral significant increases at days 7 in ROI 1 (*p<0.01), ROI 2 (*p<0.01), and the entire hemisphere (*p<0.01) for trehalose treated mice. Contralateral assessment revealed significant increases at days 7 in ROI 3 (*p<0.01) and day 7 in the entire hemisphere (*p<0.01).

Whilst two-way ANOVA and Bonferroni’s post hoc analysis revealed no overall change in Cu concentration in any region analyzed over the time course, trehalose treated mice revealed significant intra-day increases at day 7 in the ipsilateral ROI 1 “Fig 6a”, ROI 2 “Fig 6c”, and at day 7 in the entire ipsilateral hemisphere “Fig 6g” that was not observed in SSV treated mice. Similarly, at day 7 in the contralateral ROI 3 “Fig 6f” and the entire hemisphere “Fig 6h” there was a significant increase in Cu concentration. In every region for both ipsilateral and contralateral sides there was a steady increase in Cu from day seven onwards for both Trehalose treated and SSV treated mice. Whilst not significant at any single time point, Cu concentration at day 14 for every time point was higher for trehalose-treated mice over their SSV-treated counterparts. However, within the ipsilateral and contralateral ROI 1, ROI 2, ROI 3 and the entire hemispheres, the trehalose group appears to decrease in Cu concentration at the 28-day time point when compared to SSV controls.

#### Iron is modulated by trehalose after TBI

**Fig 7:**
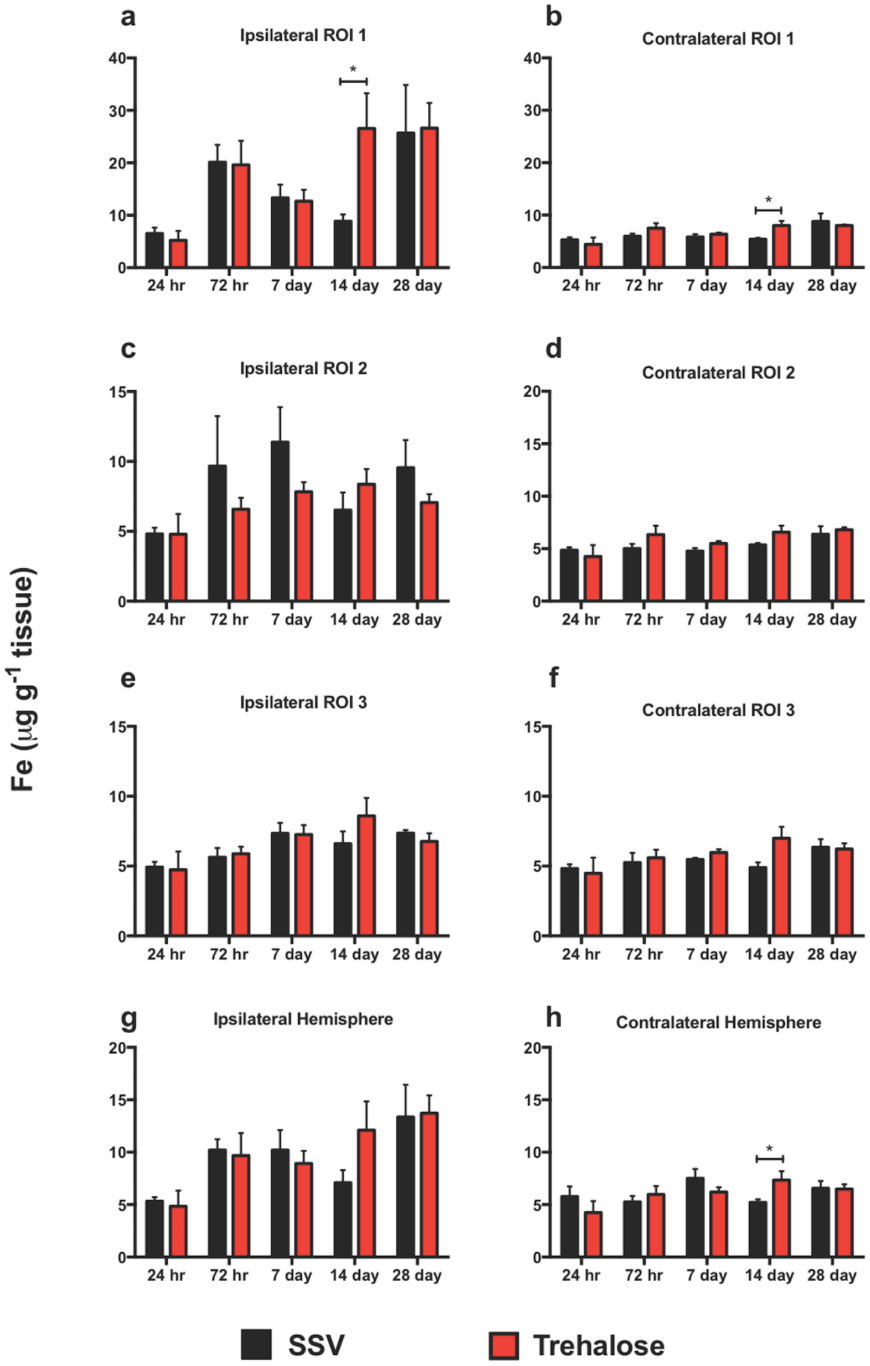
Iron is modulated across the time course. Fe was revealed to be significantly increased in the ipsilateral cortex in ROI1 at 7 days for trehalose treated mice (a) (*p<0.01). The contralateral side revealed an increase at day 14 for trehalose treated mice in ROI1 (b) (*p<0.01) and the entire hemisphere (h) (*p<0.01). Ipsilateral Fe concentrations in every region surveyed were elevated regardless of treatment when compared to equivalent contralateral regions.

There are subtle changes in the Fe profile over the time course post-injury. In every region analyzed on the ipsilateral side from day seven to fourteen there is a decrease in Fe for the SSV treated group. Conversely there is an increased trend in Fe for the trehalose treated group at the same time-points, with a significant increase observed in the ipsilateral ROI 1 at day 14 between trehalose and SSV “Fig 7a”. Comparatively, the contralateral side reflects an almost identical pattern except for ROI 2 where the SSV treated day fourteen concentrations is not decreased from day seven. However, there is a significant increase in Fe at day fourteen in the entire contralateral hemisphere “Fig 7h”. Overall, the Fe concentrations in all ipsilateral regions are greater than the contralateral regions.

### Biochemical analyses

#### Western blot analysis

**Fig 8:**
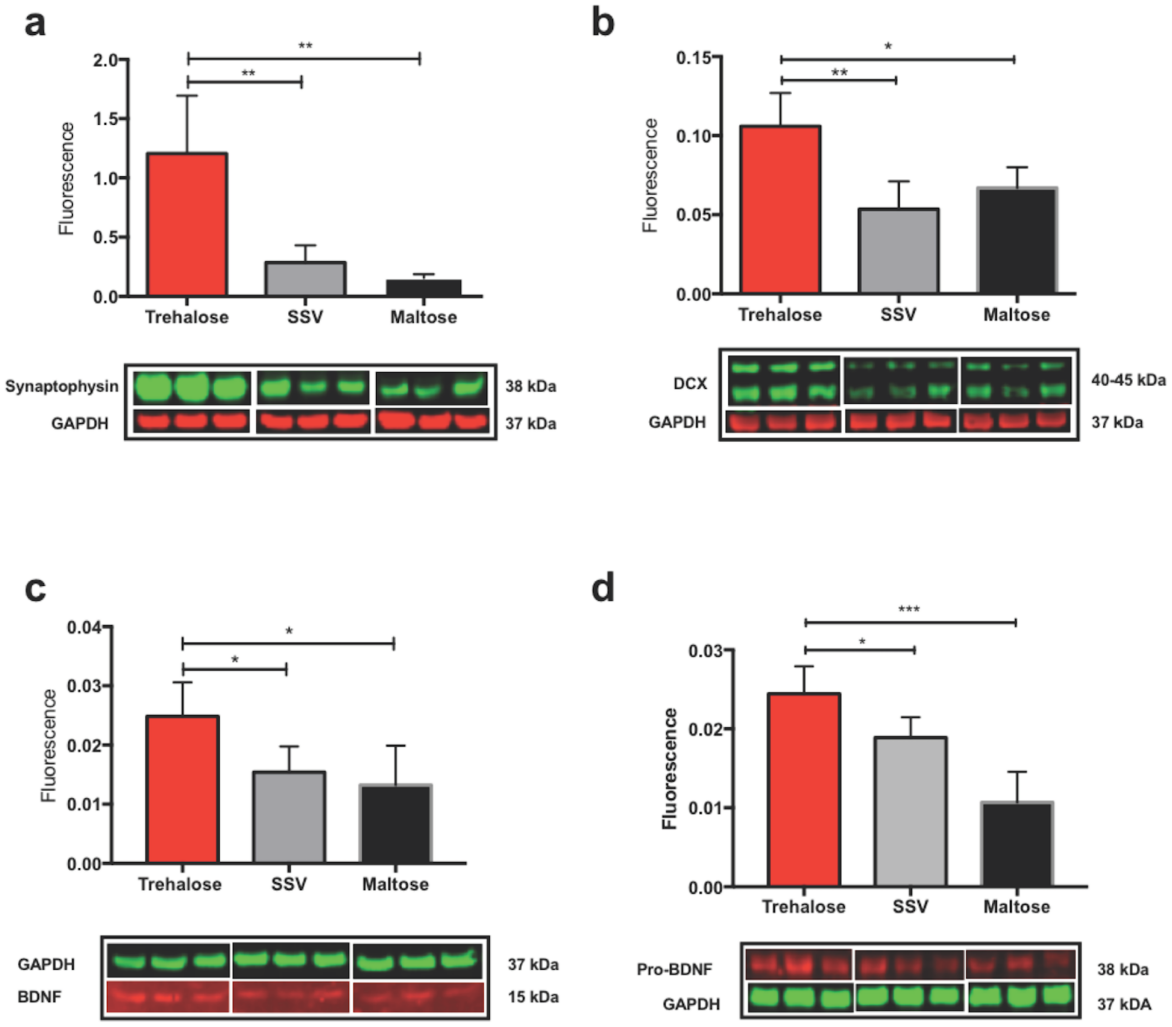
Trehalose increases synaptophysin, DCX, BDNF & Pro-BDNF in the contralateral cortex. Trehalose significantly increased synaptophysin protein in the contralateral cortex over SSV (*p<0.0221) and maltose (*p<0.0193) controls (a). Trehalose significantly increases DCX in the contralateral cortex compared to SSV (*p<0.0147) and maltose (*p<0.0123) controls (b). Trehalose also significantly increased BDNF protein in the contralateral cortex over SSV (*p<0.0296) and maltose (*p<0.0255) controls (c). Trehalose significantly increased Pro-BDNF protein in the contralateral cortex over SSV (*p<0.0210) and maltose (*p<0.0287) controls (d).

Trehalose treated mice showed a significantly elevated expression of the synaptic vesicle protein synaptophysin (a marker of synaptic activity) in the contralateral cortex when compared to SSV and maltose controls “Fig 8a”. The elevation of synaptophysin was unique to the contralateral cortex with no significant elevation observed in the contralateral hippocampus, or the ipsilateral cortex and hippocampus. However, the ipsilateral hippocampus did see a non-significant elevation of synaptophysin for the trehalose treated group when compared to control groups. Analysis of doublecortin, a neuronal migration protein that is a surrogate for neurogenesis, revealed an identical profile, whereby there was a significant increase of DCX protein observed in the contralateral cortex for trehalose treated animals “Fig 8b” and an absence of elevated DCX in the contralateral hippocampus or ipsilateral cortex and hippocampus when compared to control groups. Similarly, trehalose treated animals revealed a significant increase in both BDNF “Fig 8c” and Pro-BDNF “Fig 8d”, proteins crucial for neuronal health and synaptic remodeling, in the contralateral cortex when compared to SSV and maltose treated littermates. Both BDNF and pro-BDNF were not significantly elevated in the contralateral hippocampus or ipsilateral cortex and hippocampus.

## Discussion

In this study we have demonstrated that oral trehalose administration greatly improves cognitive outcomes, as evidenced by improved performance in the Morris water maze and Y-maze, in mice subjected to a controlled cortical impact. Additionally, performance in the open field test, which can be used to assess locomotor function and levels of anxiety/depression, was significantly improved in the trehalose treated group following TBI. These behavioural data, together with our tissue analyses, demonstrate that trehalose is significantly improving functional outcomes that are impacted as a result of brain injury. The mechanisms underlying these effects appear to most likely be driven by a previously undescribed effect of trehalose on specific pathways involved in neuronal plasticity. Given that human epidemiological data indicates that a mild single TBI is associated with an increased risk of progressive cognitive impairment [51] (which can lead to dementia [52–54]) and other psychological manifestations such as depression [55,56] and anxiety [57], our data make a compelling case for translational studies to assess the clinical benefit of trehalose treatment in patients presenting with acquired brain injury.

Based upon our previously published data in which we assessed the levels of Fe, Cu and Zn in three-month-old mice after a CCI TBI [17], in which we observed significant increases in Fe in the ipsilateral hemisphere, we hypothesized that trehalose may potentially be modulating Fe. Fe deposition is a consistent and enduring pathological consequence of TBI [17–19,58], and has also been shown to be positively related to cognitive impairment in mild traumatic brain injury [59]. However, apart from some significant time point specific observations, trehalose failed to significantly modify the overall Fe profile in any manner inconsistent with the observed Fe trends of both treatment groups. A two-way ANOVA comparison of Fe concentrations in the ipsilateral versus contralateral hemisphere of trehalose treated and SSV treateded mice revealed a virtually identical Fe concentration profile where significantly increased Fe concentrations were observed across the time course in ROI 1, ROI 2, and the entire hemisphere (data not shown). Trehalose treatment therefore did not alter the observed elevation of ipsilateral Fe concentration after TBI.

Consistent with our previously published data [17], Zn was unaltered across the time course, an observation that was unchanged with trehalose treatment. The role of Zn in TBI has been heavily scrutinized over the past several years [16,27,31,60,61], with studies indicating both protective [25,26,62] and toxic [63] roles in TBI outcomes. Although trehalose revealed no observable modulation of Zn, it should be noted that while LA-ICPMS imaging provides informative quantitation of metals on the mesoscale, it is likely that a higher resolution of analysis than the one utilized may be required to visualize the cellular translocation of Zn, that is an observed consequence of TBI [63,64].

Similarly, Cu has been proposed to play a role in TBI where its use as a serum biomarker for increased intracranial pressure after TBI has been investigated [23] and the role of Cu deficiency in the synthesis of myelin after TBI has also been proposed [15]. In this study, trehalose did not reveal any significant Cu modulating effects. Although trehalose significantly increased Cu at the seven-day assessment in the ipsilateral ROI 1, ROI 2, and ipsilateral hemisphere, as well as the contralateral ROI 3 and contralateral hemisphere, it is difficult to correlate these observations to the beneficial behavior modifications observed in the behavioral assessments employed.

The discrepancy between behavioral outcomes and the lack of any parallel changes in metal levels that might explain the functional improvement observed following trehalose treatment led us to investigate other potential mechanisms. Autophagy enhancement, a known consequence of trehalose treatment, was considered and assessed via LC3 western blot detection (data not shown). However, a significant alteration in autophagy between trehalose, SSV and maltose treated groups was expectedly not detectable, as numerous studies have shown autophagy to be naturally elevated in TBI [65–67], with one study showing LC3 greatly elevated at 32 days post TBI [68].

We therefore hypothesized that trehalose may be indirectly increasing synaptic activity and thus contributing to the behavioral performance of trehalose treated mice. Thus, we assessed the pre-synaptic vesicle protein synaptophysin and accordingly found that it was significantly increased in the contralateral cortex of trehalose treated mice (Fig 7a). There was no significant increase in the ipsilateral cortex or the ipsilateral or contralateral hippocampus. Simultaneously, we also probed for Doublecortin (DCX), a reliable and specific marker of neurogenesis [69], and revealed a significant increase in DCX protein expression in the contralateral cortex of the trehalose treated mice (Fig 7b) that was not observed in SSV treated mice.

Numerous studies indicate that in human TBI [70] and rodent models of TBI [71–73] neurogenesis can occur as part of an endogenous reparative response to traumatic injury. While the majority of cellular proliferation studies post-TBI have focused on the sub ventricular zone post-TBI [74,75], neurogenesis and increased synaptic activity in the contralateral cortex in response to injury has also been a previously observed TBI phenomena [71,73]. Moreover, studies of the role of the contralateral cortex on functional recovery in a rat model of hemiplegia [76], and a study of the plasticity of the contralateral motor cortex following focal traumatic brain injury [77], indicated a functional contribution to recovery at 14 day and five weeks, respectively.

Our data support the notion that trehalose may be inducing a neuroprotective biochemical effect in the brain post-injury. To further explore this, we chose to assess the role of BDNF and its precursor pro-BDNF. BDNF is a neurotrophic factor that promotes growth and development of immature neurons and enhances the survival and functions of adult neurons in the central nervous system [78,79]. Additionally, cortical BDNF secretion and associated dendritic growth and modulation has been clearly demonstrated to require synaptic activity [80,81], so we hypothesized that there may be a correlative increase in BDNF to coincide with the observed significant increase in the synaptic protein synaptophysin. Moreover, BDNF has been shown to be upregulated during declarative memory formation in primate cortex [82], and endogenous BDNF is required for long-term memory formation in the rat parietal cortex [83]. As hypothesized, a significant increase in BDNF and pro-BDNF expression in the contralateral cortex of trehalose treated mice was revealed, associating with both the synaptophysin and DCX protein expression results in the same region. These data indicate that trehalose has a neuroprotective role in TBI, and may further play a role in synaptic remodeling post-injury. Further studies are required to fully elucidate the biomolecular mechanisms by which trehalose exerts its effects. In particular, due to the known role of oxidative stress and neuroinflammation in disorders of the CNS, and particularly in TBI [84,85] (where, amongst other things, oxidative stress is known to impact synaptic proteins [86]), then these would be important biomarkers to examine in order to further assess the impact of trehalose on the brain, and on the brains response to injury. Furthermore, understanding how trehalose may interact with the vitagene network [87,88], which represents a group of genes that are activated in response to cellular stressors and which help maintain cellular homeostasis, would be important. Furthermore, one of the caveats of this study is that we have only examined a single dose of trehalose. Whilst trehalose is generally regarded as safe, and has been shown to be both safe and effective across a broad range of concentrations in different biological paradigms, a more thorough investigation is warranted. Specifically, completing a dose response with trehalose in this TBI paradigm, as well as pre-treatment studies, would be important to examine any potential hormetic or preconditioning effects of this compound [89]. This may be particularly relevant to situations such as chronic traumatic encephalopathy, as the apparent benefit of trehalose in the current acute TBI model may provoke considerations around the prophylactic use of trehalose for brain injuries of multiple etiologies. In such cases, therefore, it would be important to have a more complete understanding of how trehalose affected the endogenous signaling and repair pathways prior to further clinical translation. These caveats aside, it is clear that in this study trehalose yielded significantly improved behavioral outcomes post-TBI that warrant further investigation.

## Conclusion

Our results demonstrate that trehalose administration can improve cognitive outcomes in mice following brain injury. Given that trehalose is FDA “GRAS”, has a generous safety profile, and is currently used as an excipient in many pharmaceutical formulations for human use, we believe that trehalose could be a viable candidate for further pharmacological investigation as a potential therapeutic option for patients with TBI, either as a monotherapy or in conjunction with other treatment alternatives.
